# Prone Position Is Associated with Mild Cerebral Oxygen Desaturation in Elderly Surgical Patients

**DOI:** 10.1371/journal.pone.0106387

**Published:** 2014-09-12

**Authors:** Stacie Deiner, Isaac Chu, Michelle Mahanian, Hung-Mo Lin, Andrew C. Hecht, Jeffrey H. Silverstein

**Affiliations:** 1 Department of Anesthesiology, Icahn School of Medicine at Mount Sinai, New York, New York, United States of America; 2 Department of Surgery, Icahn School of Medicine at Mount Sinai, New York, New York, United States of America; 3 Department of Neurosurgery, Icahn School of Medicine at Mount Sinai, New York, New York, United States of America; 4 Department of Geriatrics and Palliative Care, Icahn School of Medicine at Mount Sinai, New York, New York, United States of America; 5 Department of Health Evidence and Policy, Icahn School of Medicine at Mount Sinai, New York, New York, United States of America; 6 Department of Orthopedic Surgery, Icahn School of Medicine at Mount Sinai, New York, New York, United States of America; 7 David Geffen School of Medicine, University of California Los Angeles, Los Angeles, California, United States of America; 8 Outcomes Research Consortium, Cleveland, Ohio, United States of America; Tokyo Metropolitan Institute of Medical Science, Japan

## Abstract

**Purpose:**

A variety of hemodynamic and respiratory alterations accompany patients in the prone position; however the effect of the prone position on intraoperative cerebral saturation has not been studied. We sought to examine whether the incidence of cerebral oxygen desaturation in elderly patients (≥68 years of age) undergoing spine surgery in the prone position was more common than patients undergoing major surgery in the supine position.

**Methods:**

We performed a retrospective cohort study of 205 patients; 63 patients underwent surgery in the prone position and 142 in the supine position. Patients were evaluated for cerebral desaturation with bilateral cerebral oximetry. The primary predictor was position, secondary were: length of the surgery, incidence and duration of cerebral desaturation episodes at several thresholds, average time of Bispectral index below threshold of 45 in minutes, average electroencephalogram suppression ratio >0, amount of blood transfused, and the incidence of hypotension and hypertension.

**Results:**

Elderly spine surgery patients in the prone position were more than twice as likely to experience mild cerebral desaturation as patients in the supine position. Patients in the prone position had longer surgeries; however cerebral desaturation in the prone position was significantly more common even when adjusted for surgery time and the occurrence of intraoperative hypotension.

**Conclusion:**

Cerebral desaturation is related to the prone position in elderly surgery patients. Future studies are necessary to determine whether this translates to a higher incidence of postoperative cognitive dysfunction and delirium.

## Introduction

The prone position causes significant physiologic changes, however its effect on cerebral physiology under anesthesia has not been well described [1]. Several physiologic alterations may place the prone patient at risk for cerebral desaturation: decrease in cerebral perfusion pressure may result from either from a decrease in mean arterial systemic pressure or an increase in intracranial pressure. However the role of prone position relative to cerebral perfusion is subject to significant equipoise because studies in ICU patients suggest improvement in cerebral perfusion pressure (secondary to improved ventilation and MAP) in the prone position while studies of prone lumbar surgery have shown a decrease in MAP which might predispose toward cerebral desaturation [2]–[4].

Cerebral oximetry is a noninvasive means of measuring the adequacy of brain oxygenation to the frontal lobes. It is currently used primarily in cardiac surgery, which is considered high risk for cerebral desaturation because of decreased cerebral perfusion on cardiopulmonary bypass [5]–[7]. Several small studies in general surgery patients have associated cerebral desaturation with postoperative cognitive dysfunction and longer PACU and hospital stays, however none of these studies have examined the effect of the prone position [8], [9].

We hypothesized that patients in the prone position are more likely to experience cerebral desaturation than patients in the supine position. If this were the case, it would be more important for patients in the prone position to maintain mean arterial pressure or to consider routine clinical use of cerebral oximetry in general noncardiac surgery patients in the prone position. In addition, if the prone position were associated with cerebral desaturation in patients under general anesthesia then it would be worthwhile to pursue outcomes studies to look at the effect of cerebral desaturation on postoperative cognitive outcomes including delirium and postoperative cognitive decline.

## Materials and Methods

The Postoperative Cognitive Function – Dexmedetomidine and Cognitive Reserve (Dexlirium Study) (R01 AG029656 – National Institute on Aging) listed on ClinicalTrials.gov NCT00561678 obtained IRB approval and informed written consent from all patients. Approval to examine the data set from the Dexlirium Study for a retrospective analysis of oxygen saturation in the prone position was provided by the Mount Sinai Program for the Protection of Human Subjects. Patient information was anonymized and de-identified prior to analysis. The present report is a retrospective analysis of a larger ongoing multi-center study, the Dexlirium Study, which is a prospective randomized trial of dexmedetomidine versus placebo in elderly surgical patients from June 2008 through January 2012. The primary study is being conducted at eight hospitals including Mount Sinai Hospital (New York), Cleveland Clinic, Englewood Hospital (New Jersey), Johns Hopkins Medical Center, Mayo Clinic, University of Maryland, Ohio State University and the University of North Carolina Hospitals.

**Figure 1:**
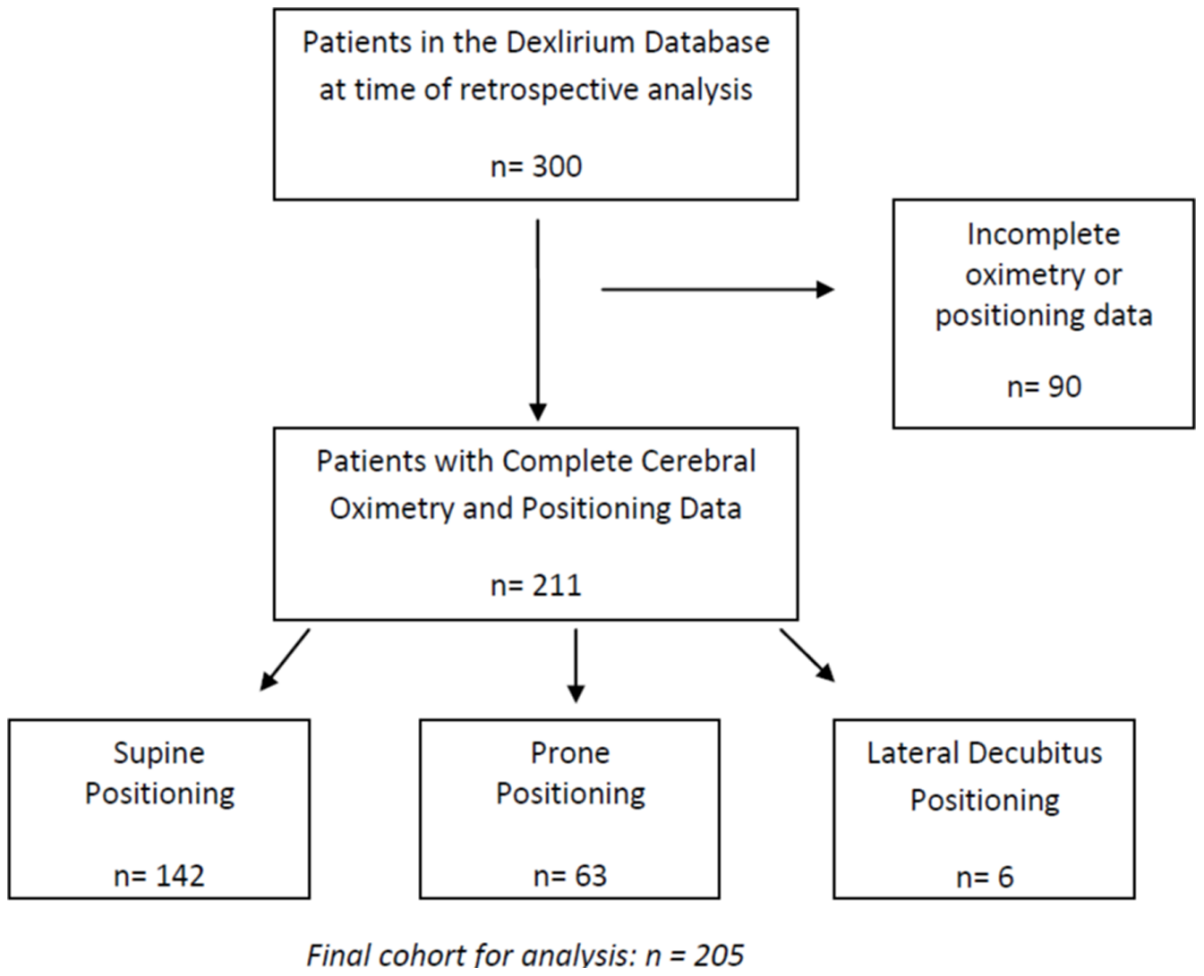
Study Population and Exclusion Criteria.

Patients over age 68, scheduled for major non-cardiac surgery under general anesthesia including general, spine, urological, or thoracic surgeries, were monitored continually throughout their operation using a cerebral oximeter. The parent study had originally recruited patients >70 years old and amended to 68 years old when recruitment goals proved difficult to meet. The data was obtained from the 6 centers that were participated from June 2008–January 2012. The parent study included patients undergoing major surgery, defined as an expected length of stay of two or more days. Inclusions and exclusion criteria are listed in Table 1. Patients were positioned as required for their surgery. At the time of the retrospective analysis, the database contained 300 patients total, patients who were scheduled for spine, general, urological, or thoracic surgeries, 90 of which were immediately excluded due to incomplete cerebral oximetry data or those who did not have cerebral oximetry monitoring which was the case at some centers in the beginning of the parent study (Figure 1). Six patients were excluded because their surgery was performed in the lateral decubitus position. Sixty-three of the patients had surgery in the prone position and 142 were supine. Patient positioning was determined from the anesthetic record. Per the study protocol clinicians were not blinded to the cerebral oximeter, although the monitor was faced away from them and they were not encouraged by the research team to respond to it. Demographics (age, gender), comorbidity (coronary artery disease, diabetes, hypertension, malignancy, renal disease, respiratory disease, history of TIA/stroke) of the supine and prone patients is shown in Table 2. The medical history was obtained from the anesthesiologist at the time of surgery. Intraoperative data included transfusion, surgery type, and the surgery duration. Anesthesia care across the six centers was guided by the following standards: avoidance of benzodiazepines and nitrous oxide, however, overall anesthetic care was based on individual clinical decision making. Processed electroencephalography (EEG) was monitored using the Bispectral Index (BIS) monitor (BIS Complete 4 Channel Monitor System, Covidien, Mansfield, MA). The parent study did not include an intervention for BIS or oximetry as this data was collected as an exploratory aim. Raw and processed EEG was recorded including time in minutes under a BIS value <45 and time in minutes during which the EEG activity indicated burst suppression, this is defined as the period in which the average burst suppression ratio >0 (minutes). To calculate the total time per case that BIS <45, we utilized the Average BIS Value (AVGBIS) which is the average of the BIS over the past minute, and summed the number of minutes from start to finish of the case that fell below a value of 45. In a similar fashion, we used the average burst suppression ratio over the past minute and summed the total number of minutes that this value fell below 0.

**Table 1:** Inclusion and Exclusion Criterion of the Parent Study.

Inclusion Criteria
Age ≥68 years
Elective major surgery under general anesthesia (>2 day hospitalization)
ASA physical status I–III
Capable and willing to consent
MMSE ≥20
Literate in English or Spanish
Noncardiac surgery

Exclusion Criteria
Cardiac surgery
Intracranial surgery
Emergency surgery
Severe visual or auditory disorder/handicaps
Illiteracy
Clinically significant Parkinson's Disease
Patients not expected to be able to complete the 3 and 6 month postoperative tests
Sick sinus syndrome without pacemaker
Hypersensitivity to alpha 2-agonists
Current 2nd (type II) or 3rd degree AV block
Major psychiatric condition (bipolar disorder, uncontrolled major depression
Schizophrenia, or dementia, does not exclude stable patients on antidepressants
ASA physical status IV or V
Clinically significant CNS disorder (e.g. history of stroke with persistent deficit)

**Table 2:** Demographics of Supine vs. Prone Groups.

Variable	Surgical Position		
	Supine (N = 142)	Prone (N = 63)	*P* Value
Age (years)	76±6	76±5	0.826
Male gender	63 (44.4%)	37 (58.7%)	0.058
CAD	16 (11.3%)	9 (14.3%)	0.542
TIA or Stroke	4 (2.8%)	5 (7.9%)	0.137
DM	23 (16.2%)	10 (15.9%)	0.954
HTN	86 (60.6%)	32 (50.8%)	0.192
Malignancy	64 (45.1%)	12 (19.0%)	<.001
Psychiatric Disorder	19 (13.4%)	11 (17.5%)	0.446
Renal Disease	8 (5.6%)	3 (4.8%)	1.000
Respiratory Disease	7 (14.2%)	5 (7.9%)	0.519
Length of Surgery (hours)	2.9 [2.0, 3.9]	3.8 [2.6, 5.4]	<.001
Baseline Cerebral Saturation %	74.1 (68.3,79.8)	74.1(69.2, 79.0)	.23
BIS<45* (min)	59 [26, 121]	114 [69, 169]	0.005
Burst supression >0* (min)	114 [69, 169]	36 [17, 60]	0.124
Needed Transfusion	14 (10.1%)	31 (50.0%)	<.001
Intraoperative Hypertension	23 (16.3%)	12 (19.4%)	0.678
Intraoperative Hypotension	59 (41.8%)	25 (40.3%)	0.878

* Sample sizes for supine and prone were 37 and 31, respectively.

Data are presented as mean (± SD), median [IQR] or N(%).

Cerebral oxygen saturation was measured using the FORESIGHT system (CAS Medical Systems, Inc., Branford, CT). The FORESIGHT system is approved by the US Food and Drug Administration as an absolute monitor, i.e. baseline readings and calculation of a change from baseline are not necessary and therefore the monitor can be placed after induction. The monitor was placed bilaterally on the forehead of each patient. The reading generated by the cerebral oximeter was the average over one minute from both sides. Monitoring was placed before the start of surgery after induction, and was recorded until the end of the surgical procedure.

The supine and prone position groups were compared by: age, presence of stroke, cancer, diabetes, hypertension, congestive heart failure, coronary artery, renal, respiratory, and psychiatric disease, baseline saturation, and intraoperative variables including case length, transfusion, the incidence of hypotension, and hypertension, and cumulative time at BIS <45 utilizing the Student's *t*-test, Wilcoxon rank sum test, and Chi Squared or Fisher's exact test where appropriate. Cerebral oximetry data were recorded at three oximetry reading thresholds (65%, 60%, and 55%) and in two ways since there is not a single “gold standard” measurement: time under the threshold (TUT) and area under the threshold (AUT) which was calculated by multiplying the difference below the threshold in percent by the time. Each oximetry value was also divided by the total surgical time to obtain time and area under threshold per hour, which normalized the data per case hours. The two position groups were compared both as median values of TUT and AUT (total and per hour) with the Wilcoxon Rank Sum test and as a binary variable of whether the subject experienced desaturation below the given threshold using the Chi-square test (Table 3). Having found that desaturation below threshold 55% was uncommon in either group; we chose to model the probability of having mild desaturation as a binary outcome defined as whether a patient's cerebral saturation ever drifted below 65%. Stepwise forward logistic regression with both entry and stay significance criteria of 0.05 was used to select predictors, which included position, age, gender, history of TIA or stroke, malignancy, had transfusion, length of the case and incidence of intraoperative hypo or hypertension. Wilcoxon rank sum test was also used to compare the total time of AUT <65% among those who had AUT <65%. Stepwise forward regression was chosen to avoid placing all variables in the model which could result in “overfitting”. Because TUT and AUT have been found to be collinear, the final model used the outcome of any incidence of desaturation below a threshold of <65%.

**Table 3:** Supine vs. Prone: Comparison of Desaturation at 3 Thresholds.

Variable	StO_2_ Threshold	Surgical Position		
		Supine* (N = 142)	Prone* (N = 63)	*P* Value
TUT	<65%	0 [0, 18.3]	2.1 [0, 107.5]	<.011
	<60%	0 [0, 0]	0 [0, 0]	0.243
	<55%	0 [0, 0]	0 [0, 0]	0.974
TUT (per hour)	<65%	0 [0, 6.7]	0.6 [0, 31.0]	0.017
	<60%	0 [0, 0]	0 [0, 0]	0.254
	<55%	0 [0, 0]	0 [0, 0]	0.988
AUT	<65%	0 [0, 25.6]	2.7 [0, 243.5]	0.010
	<60%	0 [0, 0]	0 [0, 1.2]	0.085
	<55%	0 [0, 0]	0 [0, 0]	0.683
AUT (per hour)	<65%	0 [0, 9.4]	0.7 [0, 70.0]	0.018
	<60%	0 [0, 0]	0 [0, 0.2]	0.084
	<55%	0 [0, 0]	0 [0, 0]	0.688
Experienced Desaturation	<65%	62 (43.7%)	40 (63.5%)	0.009
	<60%	23 (16.2%)	16 (25.4%)	0.122
	<55%	9 (6.3%)	4 (6.3%)	1.000

*Data are presented as median [IQR] time in minutes spent below the threshold and percent of total patients who experienced any time under the threshold.

## Results

211 patients were enrolled in the parent trial and underwent cerebral oximetry as part of the study at the time of this retrospective analysis. Six patients were excluded because they had surgery performed in the lateral decubitus position. 142 patients had surgery in the supine position and 63 had surgery in the prone position. All the patients in the prone group had lumbar or cervical spine surgery. The patients in the supine group had anterior cervical spine, general, thoracic, and urologic surgery. The two groups were similar in terms of demographic characteristics with respect to age, gender, history of coronary artery disease, diabetes mellitus, hypertension, renal disease, respiratory disease, or transient ischemic attack/stroke (Table 2). Baseline cerebral saturation was similar between the two groups. There was a significant association between positioning and history of malignancy which was more common in the supine group.

We performed univariate analyses of the relationship between intraoperative variables and the two position groups. These included: length of the surgery, TUT saturations at 65%, 60%, 55%, AUT saturations at 65%, 60%, 55%, AUT and TUT at 55%, 60%, and 65% per hour, average time of BIS below threshold of 45 in minutes, average burst suppression ratio >0 (minutes), amount of blood transfused, and the incidence of hypo- and hypertension. There was a significant difference between the prone position and the supine position with regards to median values of AUT 65, TUT 65, AUT 65 per hour, and TUT per hour. This indicates that mild desaturation (i.e. below 65 percent saturation) is more likely in the prone position. More severe desaturation indicated by the 60 percent and 55 percent thresholds was not significant. Patients in the prone position were more likely to have longer cases, more time below a BIS value of 45, and received more blood transfusions. There was no association between the incidence of hypotension or hypertension and patient position.

The incidence of severe cerebral desaturation was uncommon in our patients. Most patients spent no time under the thresholds of 55 and 60 percent. This was the same in both supine and prone patients. However, the incidence of an episode of desaturation of 65 percent was higher in patients in the prone position. Stepwise forward logistic regression identified position as the sole significant predictor for having mild desaturation (time or area below 65% >0). The odds of a mild desaturation event (AUT65) was 2.30 (95% CI: 1.24∼4.26; *p* = 0.009) times more likely for a patient in prone position vs. supine position. In the subgroup of patients who experienced any mild desaturation, the total amount of time of exposure was greater for the prone group all thresholds.

## Discussion

Our study shows that elderly patients in the prone position have more than twice the odds of experiencing an episode of mild cerebral desaturation relative to patients in the supine position. Fischer et al. found that that these mild desaturation episodes in patients undergoing aortic arch surgery (time under threshold of 60% and 65%) do have clinical significance and are associated with extended hospital stay and additional costs [6]. Kazan et al demonstrated that patients who spent time below a threshold of 65% had more than twice the incidence of non-respiratory organ failure [10]. Our study shows that mild cerebral desaturations also occur in the general surgery population and that prone patients are at higher risk. In the context of the previous outcome studies our results suggest that it may be worthwhile to compare cognitive outcomes and complications between prone and supine patients.

Our data showed that cerebral desaturation was not related to arterial hypotension. This is consistent with a recent study by Hojlund et al. which showed that the prone position results in reduced stroke volume and cardiac output due to caval compression but also with a slight increase in mean arterial pressure [11], [12]. In this study middle cerebral artery blood velocity was optimal when the patient's head was in neutral position avoiding potential occlusion to arterial flow or venous congestion. Intracranial pressure may moderate the relationship between cerebral blood flow and brain oxygenation; perfusion pressure may decline when either intracranial pressure or central venous pressure increases [13]. Although all of our patients were positioned with their head in neutral position we cannot exclude that some may have had neck compression from the foam headrest or misplaced iliac crest support.

Our study differs from the existing literature in that it uses FORESIGHT oximetry and thresholds instead of a measure of change from baseline. The FORESIGHT monitor has approval by the US FDA as an absolute monitor and time spent below even mild desaturation (<65%) thresholds have been associated with poor outcomes. Our study also contains a large group of patients which was afforded by virtue of being part of a parent study. This allowed us to focus on an exploratory aim which many studies have not had the time or funding to complete. Our study is one of the few which has focused on general surgery patients. Currently the field of cerebral oximetry is focused on cardiac surgery because of the higher incidence of morbidity and the necessity for extended measures of end organ perfusion during cardiac bypass [5]–[7]. Our study confirms that in a general surgery population, patients in the prone position may be at risk for intraoperative cerebral desaturation which could lead to adverse outcomes.

Our study has limitations; most significantly it is a retrospective analysis of an existing dataset and therefore may have confounding factors which could be limited in a randomized trial. Additionally we can only address questions based on the data collected and cannot report on clinical outcomes because the parent study has not yet been unblinded. However, this is the largest set of oximetry data ever reported on a cross section of noncardiac surgery patients and suggests that position may be an important variable for other researchers to consider in studies of postoperative cognition and outcomes. Because the study was not randomized there were some differences between the supine and prone groups which included gender, more transfusion in the prone group, and fewer surgeries for malignancy. However it is unlikely that these factors were the driving force behind the desaturation seen in the prone group because these factors became insignificant in the generation of the final model. Finally, the parent study involved randomization to receive dexmedetomidine infusion or placebo. Since the parent study has not been unblinded we do not know which patients received dexmedetomidine, however several studies suggest that dexmedetomidine has no significant effect on intracranial hemodynamic effects or metabolic parameters in humans and at most modest effects on hypocapnic animals [14]–[16].

The value of this study is that it gauges the magnitude of the difference in cerebral saturation seen between prone and supine patients. This information can be used to plan prospective studies using either crude (e.g. Mini Mental Status) or in-depth cognitive outcomes to see whether the difference we found has clinical relevance. Most likely poor cognitive outcomes in elderly patients after non-cardiac surgery are a multi-factorial problem. Elderly patients for spine surgery in the prone position are ever increasing in number, and improvement in their postoperative outcomes has major clinical and social implications. Our study suggests that cerebral desaturation and patient positioning during surgery are important variables which should be measured in outcomes studies of this population especially when examining postoperative cognition.
